# SCFA Treatment Alleviates Pathological Signs of Migraine and Related Intestinal Alterations in a Mouse Model of NTG-Induced Migraine

**DOI:** 10.3390/cells10102756

**Published:** 2021-10-14

**Authors:** Marika Lanza, Alessia Filippone, Alessio Ardizzone, Giovanna Casili, Irene Paterniti, Emanuela Esposito, Michela Campolo

**Affiliations:** Department of Chemical, Biological, Pharmaceutical and Environmental Sciences, University of Messina, Viale Ferdinando Stagno D’Alcontres, 31-98166 Messina, Italy; mlanza@unime.it (M.L.); afilippone@unime.it (A.F.); aleardizzone@unime.it (A.A.); gcasili@unime.it (G.C.); ipaterniti@unime.it (I.P.); campolom@unime.it (M.C.)

**Keywords:** central nervous system, enteric nervous system, migraine, short-chain fatty acids, sodium propionate, sodium butyrate

## Abstract

Background: There is a growing realization that the gut–brain axis signaling is critical for maintaining the health and homeostasis of the Central Nervous System (CNS) and the intestinal environment. The role of Short-Chain Fatty Acids (SCFAs), such as Sodium Propionate (SP) and Sodium Butyrate (SB), has been reported to counteract inflammation activation in the central and Enteric Nervous System (ENS). Methods: In this study, we evaluated the role of the SCFAs in regulating the pathophysiology of migraine and correlated dysregulations in the gut environment in a mouse model of Nitroglycerine (NTG)-induced migraine. Results: We showed that, following behavioral tests evaluating pain and photophobia, the SP and SB treatments attenuated pain attacks provoked by NTG. Moreover, treatments with both SCFAs reduced histological damage in the trigeminal nerve nucleus and decreased the expression of proinflammatory mediators. Ileum evaluation following NTG injection reported that SCFA treatments importantly restored intestinal mucosa alterations, as well as the release of neurotransmitters in the ENS. Conclusions: Taken together, these results provide evidence that SCFAs exert powerful effects, preventing inflammation through the gut–brain axis, suggesting a new insight into the potential application of SCFAs as novel supportive therapies for migraine and correlated intestinal alterations.

## 1. Introduction

Migraine is a severe and disabling brain condition characterized by recurrent episodes of headache [1]. It is a complex and multifactorial brain disorder [1] with a higher prevalence in females (17.5%) than in males (8.9%) [1,2]. Several risk factors have been associated with migraine including genetic factors, gender, and age [3]. Clinically, migraine manifests itself with often one-sided pain, intolerance to light, and vomiting [1]. The exact pathophysiology of migraine is unclear, but it is believed that activation of the Trigeminovascular System (TGVS) and Cortical Spreading Depression (CSD) play an important role in the pathophysiology of migraine [3,4]. Scientific evidence has also revealed that inflammation and oxidative stress play a key role in migraine development [5,6,7]. It is thought that inflammation of the CNS, also known as neuroinflammation, is directed by the release of vasoactive proinflammatory factors [4,8], while oxidative stress is implicated in migraine disorder due to an imbalance between the production of Reactive Oxygen Species (ROSs) and the reduction of antioxidant defense mechanisms, causing oxidative damage to DNA, lipids, and proteins [6]. There may be an association between migraine and the gut environment. Sickness and heaving are frequently connected with migraine attacks. Additionally, research suggests that individuals with consecutive migraine episodes might be bound to foster gastrointestinal issues. Thus, most of the studies aiming to investigate the roles of gut resident intestinal flora in neurological disorders usually use microbiota estimation, but also delineating the immune cells and their inflammatory mediators release neurotransmitter role in the Enteric Nervous System (ENS) [9]. The brain normally regulates the movements and functions of the GI tract (sensory and secretion), and strategies targeting any dysfunctions through the central and ENS are needed. Thus far, different approaches have been suggested including probiotic and vitamin supplementation in the diet [10], but the treatment for acute migraine using triptans, in particular sumatriptan, belonging to Analgesic and Nonsteroidal Anti-Inflammatory Drugs (NSAIDs), is still encouraged for migraine attacks [4,11]. Although sumatriptans were the first successful treatment for migraine attacks, currently, there are still no very effective and widely applicable drug treatments for migraine management; therefore, the study and development of more effective and safe antimigraine agents are needed. Recent studies demonstrated that Sodium Butyrate (SB) and Sodium Propionate (SP) exert anti-inflammatory and neuroprotective effects in various disorders [12,13]. SB and SP are natural Short-Chain Fatty Acids (SCFAs) present in the diet and produced in the colon by the anaerobic fermentation of undigested carbohydrates [14]. Through microbiota metabolites, including SCFAs, it has been recently reported that the gut participates in the regulation of several systems [15]. Among the SCFAs, the anti-inflammatory properties of SB and SP have been shown in an increasing number of in vivo and in vitro models of inflammatory diseases, despite the exact mechanism of action not being entirely understood [16,17,18]. Scientific evidence has revealed that both SB and SP inhibit Histone Deacetylases (HDACs), resulting in the hyperacetylation of core histone proteins (H3 and H4) expressed by some inflammatory-related genes [15], and the Nuclear Factor kappa-light-chain-enhancer of activated B cells’ (NF-κB) translocation, which is a well-known inflammatory mediator, reducing consequently inflammatory cascade activation and oxidative stress [15,16,19]. Since SB and SP possess important effects as neuromodulators of the CNS [20], repairing spinal cord injured tissue and reducing neutrophils, as well as reporting antioxidant properties, counteracting ROS production [12,18], it follows that the activity of the sympathetic nervous system is influenced by SCFAs’ metabolism, as stated by the SP and SB interactions with G-Protein-coupled Receptors (GPRs), such as GPR41 and GPR43 of the ganglia of the ENS [20]. Thus, SB and SP have insightful effects also on gut functionality and health, due to the Free Fatty Acid 2 (FFA2) and Free Fatty Acid 3 (FFA3) receptors bounding, causing the suppression of intestinal inflammation and supporting the maintenance of intestinal homeostasis [14,21,22]. Therefore, on the basis of this scientific evidence, the aim of this study was to evaluate the beneficial effects of SB and SP in the brain, as well as their impact on the gut–brain axis in an in vivo model of Nitroglycerine (NTG)-induced migraine, suggesting a new insight into the potential application of SCFAs for a multi-organ disease.

## 2. Materials and Methods

### 2.1. Animals

CD1 adult mice (females, 25 to 30 g, Envigo, Casatenovo, Lecco, Italy) were housed in a controlled environment (22 ± 2 °C, 55 ± 15% relative humidity, 12 h light/dark cycle). Standard diet and tap water were available ad libitum. Animal care followed Italian regulations on the protection of animals used for experimental and other scientific purposes (Ministerial Decree 16192), as well as the Council Regulation (EEC) (Official Journal of the European Union L 358/112/18/1986). All compounds were obtained from Sigma-Aldrich Company Ltd. (Milan, Italy) and Bio-Optica Spa Company (Milan, Italy). All stock solutions were prepared in nonpyrogenic saline (0.9% NaCl; Baxter, UK).

### 2.2. Migraine Model Induction

NTG was prepared from a stock solution of 5.0 mg/mL nitroglycerin in 30% alcohol, 30% propylene glycol, and water (American Regent). The dose of NTG used was 10 mg/kg diluted in 0.9% saline [23]. NTG should be prepared fresh for each test day. All injections were administered as a 10 mg/kg volume, and the vehicle used in these experiments was 0.9% saline. Animals were treated orally with SP and SB at doses of 10 mg/kg, 30 mg/kg, and 100 mg/kg, 5 min following NTG injection. Mice were sacrificed 4 h following NTG injection; the whole brain with the rostral spinal cord was removed for analysis.

#### Experimental Groups

Animals were randomly divided into the following groups:

- Group sham + vehicle (veh): mice received saline;
- Group NTG: mice received NTG (10 mg/kg) intraperitoneally;
- Group NTG + sumatriptan: mice received sumatriptan orally (600 μg/kg) 5 min after NTG (10 mg/kg) intraperitoneally;
- Group NTG + SP 10 mg/kg: mice received SP orally at a dose of 10 mg/kg 5 min after NTG injection;
- Group NTG + SP 30 mg/kg: mice received SP orally at a dose of 30 mg/kg 5 min after NTG injection;
- Group NTG + SP 100 mg/kg: mice received SP orally at a dose of 100 mg/kg 5 min after NTG injection;
- Group NTG + SB 10 mg/kg: mice received SB orally at a dose of 10 mg/kg 5 min after NTG injection;
- Group NTG + SB 30 mg/kg: mice received SB orally at a dose of 30 mg/kg 5 min after NTG injection;
- Group NTG + SB 100 mg/kg: mice received SB orally at a dose of 100 mg/kg 5 min after NTG injection.

The minimum number of mice for every technique was estimated with the statistical test “ANOVA: Fixed effect, omnibus one-way” with the G-power software. This statistical test generated a sample size equal to *n* = 10 mice for each technique.

Data regarding the groups of control mice (sham+ SP 10 mg/kg, sham+ SP 30 mg/kg, sham+ SP 100 mg/kg, group sham+ SB 10 mg/kg, sham+ SB 30 mg/kg, and sham+ SB 100 mg/kg) are not shown because SP and SB alone demonstrated no significant histological changes. The doses of SP and SB were based on a previous dose–response study in our laboratory [12,13,18]. The dose of sumatriptan was used as previously described by Ferrari MD and colleagues [24].

### 2.3. Behavioral Tests

#### 2.3.1. Tail Flick Test

The tail flick test as an acute model of pain assesses the antinociceptive effect of drugs by measuring the latency time [25]. Latency time is the time from the onset of heat exposure to withdrawal of the tail [25]. The water temperature in 250 mL beakers was maintained at 46 ± 0.1 °C using a hot plate or at 15 ± 0.1 °C using crushed ice. For testing, each mouse was wrapped in a terry cloth towel and its tail submerged 5 cm. Latency to flick or curl the tail was recorded with a 40 s cutoff, as described by Sufka et al. [26].

#### 2.3.2. Orofacial Formalin Test

The orofacial formalin test was performed as previously described [26]. The CD1 mice were acclimatized to the laboratory environment for at least 1 h before use. The mice received a subcutaneous injection of 20 µL of diluted formalin (as the formalin model group) or saline (sham group) into the center of the right vibrissa pad. Solutions were prepared from commercially available stock formalin (an aqueous solution of 37% formaldehyde) and further diluted in isotonic saline to 4%. SP and SB (40 μL for 10 mg/kg, 30 mg/kg, and 100 mg/kg) were injected intraperitoneally 30 min before formalin injection. The mice did not have access to food or water during the test. After injection, the animals were immediately placed back in the test box for a 45 min observation period. The observation period was divided into 15 blocks of 3 min, and the number of seconds the animal spent in ipsilateral face rubbing or grooming was measured during Phase I (0–12 min) and Phase II (12–45 min) of formalin-induced pain, as previously described by Raboisson et al. [27].

#### 2.3.3. Hot Plate Test

The hot plate test was performed by placing the mice on a hot plate at 50 °C. The response time for observed behavioral changes such as paw licking, stomping, jumping, and escaping from the hot plate was as previously described [28]. The latency time to pain reaction was measured at 30 min, 60 min, 90 min, 120 min, and 240 min post NTG injection.

#### 2.3.4. Light/Dark Test

The light/dark test was performed to quantify by the “The International Classification of Headache Disorders, 3rd edition” (ICHD-3) criteria of photophobia and reduced activity associated with migraine [29]. The typical light/dark box had two compartments connected to each other with an opening. The mice were placed in the light chamber first, and the behavior of the animal was recorded over a 5–10 min period. The latency of the first entry into the dark compartment, the percentage of time spent in the light and dark compartments, and the number of dark to light transitions were quantified [29].

#### 2.3.5. Histological Analysis

Mice were sacrificed after 4 h of NTG injection, and the brain and the intestine tissues were processed for histological analysis. Sections were then deparaffinized and stained with hematoxylin and eosin (H&E) [13]. All sections were analyzed by a pathologist using an Axio vision Zeiss microscope (Milan, Italy).

#### 2.3.6. Western Blot Analysis of COX2 and iNOS

Western blot analysis was performed on the whole brain with the rostral spinal cord tissues harvested 4 h after NTG injection, as previously described by Casili et al. [30]. Tissues from each mouse were homogenized to extract the cytosolic and nuclear fraction. Protein concentrations were calculated by the Bio-Rad protein assay using bovine serum albumin as the standard. Briefly, samples were heated at 100 °C for 5 min, and equal amounts of protein were separated on 18% SDS-PAGE gel and transferred to a nitrocellulose membrane. Then, the membranes were blocked with 5% (*w*/*v*) nonfat dried milk in buffered saline (PM) for 45 min at room temperature and subsequently probed with specific antibodies: anti-COX2 (1:500; Santa Cruz Biotechnology, Dallas, TX, USA sc-376861) and anti-iNOS (1:500; 610432 BD Transduction) in 1× PBS, 5% *w/v* nonfat dried milk, and 0.1% Tween-20 (PMT) at 4 °C overnight. The membranes were incubated with peroxidase-conjugated bovine antimouse Immunoglobulin G (IgG) secondary antibody or peroxidase-conjugated goat antirabbit IgG (1:2000, Jackson ImmunoResearch, West Grove, PA, USA) for 1 h at room temperature. To establish that the blots were loaded with equal amounts of proteins, they were also incubated in the presence of the antibody against the β-actin protein (cytosolic fraction, 1:500; Santa Cruz Biotechnology) or lamin A/C (nuclear fraction, 1:500 Santa Cruz Biotechnology). Signals were revealed with the Enhanced Chemiluminescence (ECL) detection system reagent according to the manufacturer’s instructions (Thermo, Waltham, MA, USA). The relative expression of the protein bands was quantified by densitometry with the Bio-Rad ChemiDocTMXRS + software and standardized to β-actin levels, as an internal control.

#### 2.3.7. Immunohistochemical Localization of Tumor Necrosis Factor, Interleukin-1β, and Neuronal Nitric Oxide Synthase in the Intestine

After deparaffinization and rehydration, endogenous peroxidase was removed with 0.3% H_2_O_2_ in 60% methanol for 30 min. Nonspecific adsorption was minimized by incubating the section in 2% normal goat serum in PBS for 20 min. The intestine sections were then incubated overnight with primary IL-1β (Santa Cruz Biotechnology; 1:100 in PBS), TNF (Santa Cruz Biotechnology; 1:100 in PBS), and nNOS (Santa Cruz Biotechnology; 1:100 in PBS). Sections were washed with PBS and incubated with peroxidase-conjugated bovine antimouse IgG secondary antibody or peroxidase-conjugated goat antirabbit IgG (1:2000 Jackson Immuno Research, West Grove, PA, USA). Specific labeling was detected with a biotin-conjugated goat antirabbit IgG or biotin-conjugated goat antimouse IgG and avidin–biotin peroxidase complex (Vector Laboratories, Burlingame, CA, USA). To verify the binding specificity for TNF, IL-1β, and nNOS, control sections were also incubated with only the primary antibody (no secondary) or with only the secondary antibody (no primary). In these controls, no positive staining was found in the sections, indicating that the immunoreaction was positive in all the experiments. The immunohistochemical pictures were collected by a Zeiss microscope using the Axio Vision software (White Palins, New York, NY, USA) For the graphic display of the densitometric analyses, the percentage of positive staining (brown staining) was measured by computer-assisted color image analysis (Leica QWin V3, Cambridge, UK). The percentage area of immunoreactivity (determined by the number of positive pixels) was expressed as the percentage of total tissue area (red staining) within five random fields at 20× magnification. In particular, firstly, the colors of the images that were stained to the molecule of interest were defined. Once these colors were defined, they were automatically detected in all samples. This is a semiquantitative analysis that measures areas and not intensities [19,20,21]. In particular, the densitometry analysis was carried out on a section in which the ileum tissues were orientated longitudinally in order to observe all the histological portions.

#### 2.3.8. Immunofluorescence Localization of Brain-Derived Nerve Factor and Neurotrophin-3 in the Intestine

After deparaffinization and rehydration, the detection of BDNF and NT3 was carried out after boiling sections in 0.1 M citrate buffer for 1 min. Nonspecific adsorption was minimized by incubating in 2% (*v*/*v*) standard goat serum in PBS for 20 min. The ileum sections were incubated overnight with murine monoclonal anti-BDNF antibodies (1:100, Santa Cruz Biotechnology, Santa Cruz, CA, USA) at 37 °C in a humidified oxygen and nitrogen chamber. Sections were then incubated with a secondary antibody—a Fluorescein-Isothiocyanate (FITC)-conjugated antimouse Alexa Fluor-488 antibody (1:2000 *v/v* Molecular Probes, UK)—for 1 h at 37 °C. Nuclei were stained by adding 2 µg/mL 40, 60-Diamidino-2-Phenylindole (DAPI; Hoechst, Frankfurt, Germany) in PBS. Sections were observed at 20× magnifications by a Leica DM2000 microscope (Leica, Milan, Italy). Optical sections of samples were obtained by an HeNe laser (543 nm), a UV laser (361–365 nm) and an argon laser (458 nm) at a 1 min, 2 s scanning rapidity with up to 8 averages; 1.5 µm sections were attained using a pinhole of 250. Examining the most luminously labeled pixels and using settings that allowed clear visualization of the structural details, while keeping the maximum pixel intensities close to 200, established the contrast and brightness. The same settings were used for all images obtained from the other samples that were processed in parallel. Digital images were cropped and figure montages produced using Adobe Photoshop 7.0 (Adobe Systems; Palo Alto, CA, USA).

#### 2.3.9. ELISA Kit Assay

ELISA assays were performed as previously described by Campolo et al. [31]. TNF and IL-1β levels were measured in supernatants by the ELISA kit Invitrogen Thermo Fisher Scientific (Waltham, MA, USA), according to the manufacturer’s instructions.

#### 2.3.10. Real-Time Quantitative PCR Amplification

RT-qPCR analysis was executed as previously reported [32].

The amplified PCR products were quantified through the calculated Cycle Thresholds (CTs) of target genes and β-actin mRNA. RTqPCR was performed for the evaluation of the following gene expressions: IL-6 (forward: 5′-GCGGTAAAGGCATGGATAT-3′, reverse: 5′-GTTGTAGTTGGAAGGGCAG-3′) and IL-8 (forward: 5′-CGGCAATGAAGCTTCTGTAT-3′, reverse: 5′-CCTTGAAACTCTTTGCCTCA-3′). After normalization, the mean value of the normal sham target levels was chosen as the calibrator, and the results were expressed according to the 2−∆∆Ct method such as the fold change relative to the normal sham.

#### 2.3.11. Statistical Evaluation

All values are indicated as the mean ± Standard Error of the Mean (SEM) of N observations. N represents the number of animals engaged. The experiment is descriptive, as a minimum of three experiments were performed on different days on tissue sections collected from all animals in each experimental group. Data were analyzed with the GraphPad Prism software, by one-way ANOVA followed by a Bonferroni post hoc test for multiple comparisons. A *p*-value of less than 0.05 was considered significant.

## 3. Results

### 3.1. SCFA Treatments Reduced NTG-Induced Hyperalgesia and Pain

**Figure 1:**
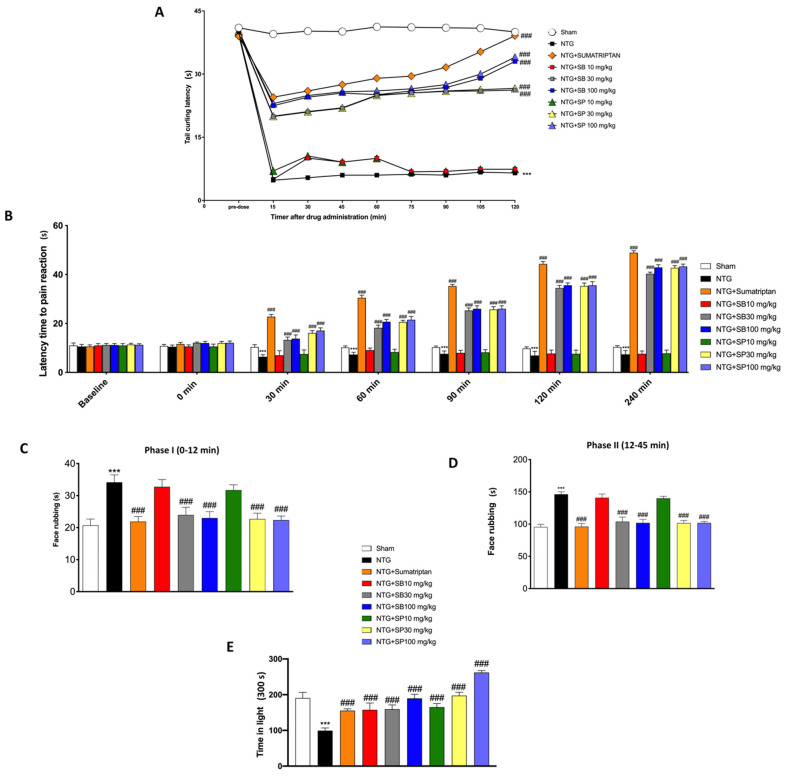
SCFA treatments reduces NTG-induced hyperalgesia and pain. NTG injection considerably decreases tail flick latency compared to sham mice (**A**). SCFA treatment of 30 mg/kg and 100 mg/kg significantly increases tail flick latency (**A**) and significantly increases latency time for pain reaction already after 30 min following NTG injection (**B**). NTG administration considerably increases the total time of rubbing in Phases I and II of the orofacial formalin test compared to the sham group. The highest doses of SCFA treatments meaningfully reduces face rubbing time in both phases (**C**,**D**). Time in light exposure decreases in NTG-injected mice, compared to the sham group (**E**), while the treatment with SCFAs significantly reduces photophobia (**E**). Data are representative of at least three independent experiments. One-way and two-way ANOVA test. *** *p* < 0.001 vs. sham; ^###^ *p* < 0.001 vs. NTG. *N* = 10 mice/group for each technique.

NTG-evoked hyperalgesia in mice was developed as a model for sensory hypersensitivity associated with migraine. The tail flick test is a thermal hyperalgesia test in which the tail of the animal is subjected to a warm source, retracting the tail (“tail flick”) when the situation becomes painful. In this study, it was shown that the treatment with both SCFAs at doses of 30 mg/kg and 100 mg/kg significantly increased tail flick latency, suggesting an SCFA-mediated antinociceptive effect (Figure 1A). SCFA treatments at both doses (30 mg/kg and 100 mg/kg), but not 10 mg/kg, significantly increased the latency time for pain reaction related to the increase in time from 0 min (starting time of NTG injection) up to 240 min; furthermore, sumatriptan treatment, as the negative control, increased the latency time to pain even more (Figure 1B). In the orofacial formalin test, total time spent in face rubbing evoked by formalin injection was counted in Phases I (Figure 1C) and II (Figure 1D) of the tests. NTG administration significantly increased the total time of rubbing in Phases I and II of the formalin test, while SCFA administration, at both doses of 30 mg/kg and 100 mg/kg, significantly reduced the nociceptive score (face rubbing time) in Phases I and II of the orofacial formalin test (Figure 1C,D). The symptoms of migraine headache are intensified during exposure to light; in fact, migraine photophobia is experienced by nearly 90% of migraine sufferers with normal eyesight and depends on the photic signals from the eye that converge on trigeminal vascular neurons somewhere along their path [30]. In this study, we showed that NTG injection causes restlessness in mice, and contrarily, SCFA-treated mice with higher doses of 30 mg/kg and 100 mg/kg were less susceptible to light (Figure 1E).

### 3.2. NTG-Induced Neurodegeneration in Trigeminal Nucleus Is Attenuated by SCFA Treatments

**Figure 2:**
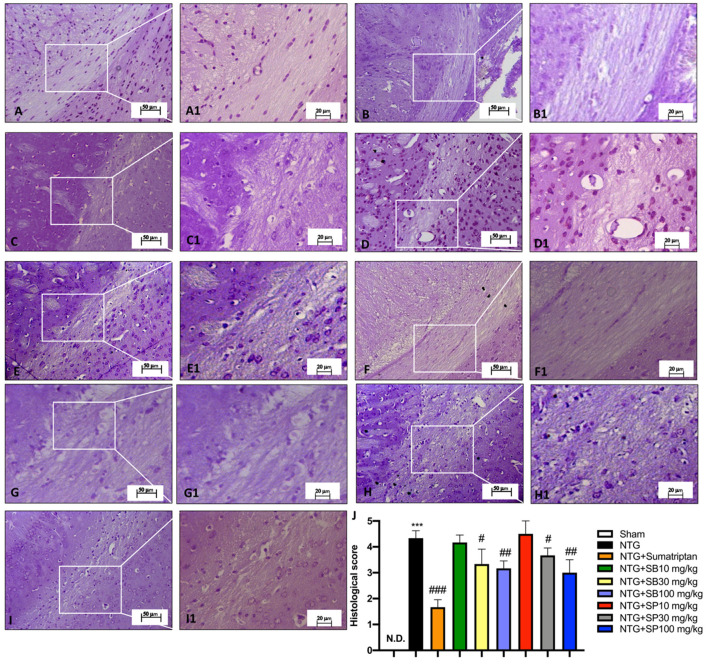
NTG-induced neurodegeneration in the trigeminal nucleus is attenuated by SCFA treatments. Cresyl violet staining shows alterations of the SpVC area in NTG-injected mice (**B**,**B1**,**J**) compared to the sham group (**A**,**A1**,**J**). Sumatriptan administration significantly reduces NTG damage in mice (**C**,**C1**,**J**). SCFA treatment, at the highest doses (**E**,**E1**,**F**,**F1**,**H**,**H1**,**I**,**I1**,**J**), appreciably restores the trigeminal neurons of the SpVC area in a more effective way than SCFAs at a dose of 10 mg/kg (**D**,**D1**–**G**,**G1**,**J**). Data are representative of at least three independent experiments. One-way ANOVA test. N.D.: Not Detectable; *** *p* < 0.001 vs. sham; ^#^ *p* < 0.05 vs. NTG; ^##^ *p* < 0.01 vs. NTG; ^###^ *p* < 0.001 vs. NTG. *N* = 10 mice/group for each technique.

The symptoms that appear before the onset of migraine are related to abnormal neuronal activity in cortical and brainstem structures; in particular, it is widely accepted that trigeminal sensory information can reach the hypothalamus via multisynaptic pathways through the brainstem [33]. The perception of trigeminal pain is mainly modulated in lamina V of the Spinal trigeminal nucleus (SpV) [34]. Thus, to define the NTG-induced alterations of the SpVC area, the brain was stained with cresyl violet, from which significant neuronal damage in NTG-injured mice was observed (Figure 2A) compared to the sham and sham + sumatriptan groups (Figure 2B,C, respectively). On the contrary, the treatment with SCFAs, mainly at the doses of 30 mg/kg and 100 mg/kg (Figure 2E,F,H,I; see the histological score, Figure 2J), significantly ameliorated the cytoarchitecture of the SpVC area, better than SCFAs at a dose of 10 mg/kg (Figure 2D,G, respectively; see the histological score, Figure 2J), restoring a large number of trigeminal neurons.

### 3.3. The Effects of SCFAs on the Anti-Inflammatory Pathway in NTG-Induced Migraine

**Figure 3:**
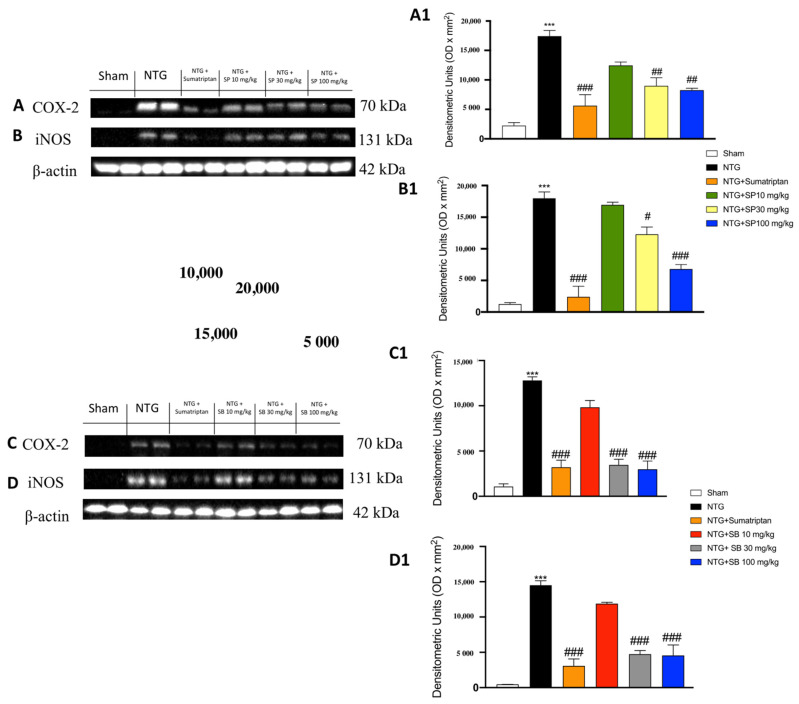
SCFAs administration reduces pro-inflammatory enzymes in NTG-injected mice. Western blot analysis of iNOS and COX-2 shows an increased expression in the NTG groups compared to the sham animals (**A**,**A1**,**B**,**B1**,**C**,**C1**,**D**,**D1**). SCFAs of 10 mg/kg are not able to reduce the expression of COX-2 and iNOS; SCFAs at the two highest doses significantly reduce these expressions as well as sumatriptan administration (**A**,**A1**,**B**,**B1**,**C**,**C1**,**D**,**D1**). Data are representative of at least three independent experiments; one-way ANOVA test. *** *p* < 0.001 vs. sham; ^#^ *p* < 0.05 vs. NTG; ^##^ *p* < 0.01 vs. NTG; ^###^ *p* < 0.001 vs. NTG. *N* = 10 mice/group for each technique.

To verify the anti-inflammatory activity of SCFAs, the levels of COX-2 and iNOS were quantified in the cytosolic fraction. COX-2 and iNOS antibodies showed basal expression in the sham groups, which was significantly increased in the NTG group (Figure 3A,B: see the densitometry analyses, Figure 3A1,B1 for SP; Figure 3C,D: see the densitometry analyses Figure 3C1,D1 for SB). Treatment with SCFAs of 10 mg**/**kg did not show any significant reduction in the iNOS and COX-2 levels, whereas this expression was markedly reduced following the treatment with SCFAs at a dose of 30 mg**/**kg and even more at a dose of 100 mg**/**kg (Figure 3A,B: see the densitometry analyses Figure 3A1,B1 for SP; Figure 3C,D: see the densitometry analyses Figure 3C1,D1 for SB).

### 3.4. SCFA Treatments Attenuate Intestinal Alterations following NTG Injection

**Figure 4:**
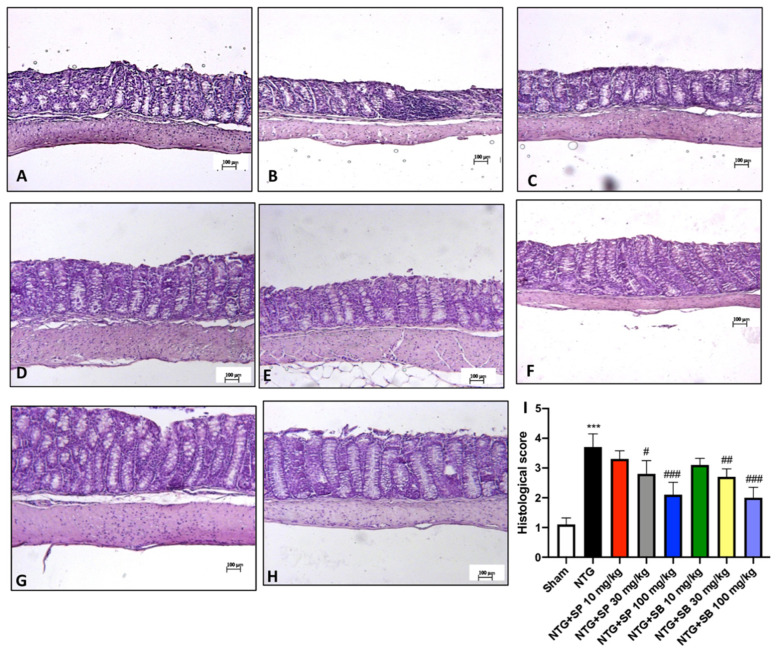
SCFA treatments attenuate intestinal alterations in NTG-injected mice. H&E staining shows an inflammatory condition in NTG animals (**B**,**I**) compared to the sham group (**A**,**I**). SCFA administration (**D**,**E**,**G**,**H**,**I**) at the highest doses effectively improves histological damage due to NTG injection. Treatments with SCFAs of 10 mg/kg are ineffective (**C**,**F**,**I**). Data are representative of at least three independent experiments; one-way ANOVA test. *** *p* < 0.001 vs. sham; ^#^ *p* < 0.05 vs. NTG; ^##^ *p* < 0.01 vs. NTG; ^###^ *p* < 0.001 vs. NTG. *N* = 10 mice/group for each technique.

Ileum sections were stained with H&E for mucosal damage and neutrophil infiltration evaluation. The histological analysis revealed a prominent inflammatory response and the loss of the regular intestinal architecture in NTG-injected mice compared to the control mice (Figure 4A,B, respectively; see the histological score, Figure 4I), indicating that the stimulation of SNC following NTG injection affects the intestinal microenvironment. The histopathological changes in the structure of intestinal mucosa were significantly ameliorated by the intraperitoneally injection of 30 mg/kg and 100 mg/kg of SCFAs (Figure 4D,E for SP; Figure 4G,H for SB; see the histological score, Figure 4I), denoting a reduction of the intestinal injury provoked by NTG-induced migraine injection. However, a low dose of SCFAs of 10 mg/kg did not show a significant difference from the NTG mice (Figure 4C,F; see the histological score, Figure 4I).

### 3.5. Increased Cytokine Release Is Reduced by SCFA Treatments following NTG-Induced Migraine in the Intestine

**Figure 5:**
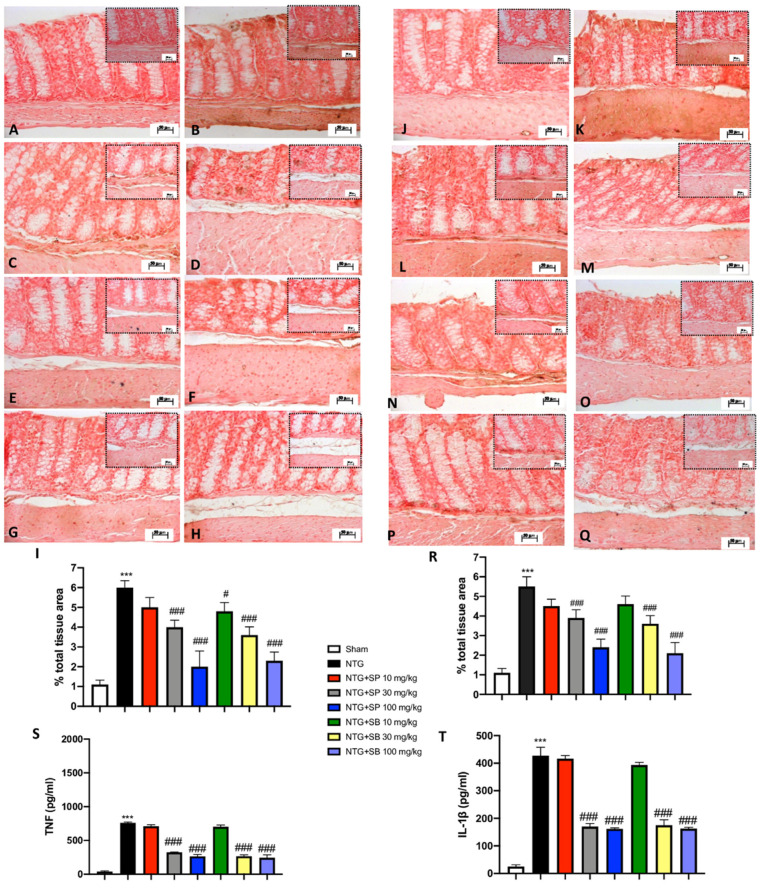
SCFA treatments reduce TNF and IL-1β expression following NTG injection. NTG-injected mice show positive immunostaining for TNF and IL-1β (**B**,**I**;**K**,**R**, respectively), compared to the sham animals (**A**,**I**;**J**,**R**, respectively). SB of 10 mg/kg slightly reduces positive immunostaining for TNF (**F**,**I**). SCFAs of 30 mg/kg and 100 mg/kg strongly decrease cytokine expression following NTG administration (**D**,**E**,**G**,**H**,**I**,**M**,**N**,**P**,**Q**,**R**, respectively). Other oral treatments do not show any significant downregulation of TNF and IL-1β expression (**C**,**I**,**L**,**O**,**R**). Quantification of cytokines TNF and IL-1β (**S**,**T**) quantities using KIT ELISA. Data are representative of at least three independent experiments; one-way ANOVA test. *** *p* < 0.001 vs. sham; ^#^ *p* < 0.05 vs. NTG; ^###^ *p* < 0.001 vs. NTG. *N* = 10 mice/group for each technique.

The integrity of intestinal mucosal homeostasis is critical for gut health since the dysfunction of its integrity associated with cytokine release occurs during inflammatory diseases [35]. Thus, to characterize the inflammatory state activated following NTG injection, we performed Immunohistochemistry (IHC) on ileum sections for TNF and IL-1β. The ileum of the NTG group showed a higher percentage of labeled epithelial cells for TNF compared to the control mice (Figure 5A,B, respectively). After SCFA administration to NTG-injected mice, we noticed a reduction of intestinal positive staining for TNF. In particular, SCFA treatments at both doses of 30 mg/kg and 100 mg/kg significantly downregulated cytokine release (Figure 5D,E for SP; Figure 5G,H for SB), despite no significant differences found in mice treated with 10 mg/kg of SCFAs (Figure 5C,F for SP and SB, respectively). Similarly, the intestinal damage following NTG-induced migraine was indicated by increased staining of the IL-1β marker across the intestinal layers, compared to the control mice (Figure 5K,J, respectively). A decrease of IL-1β immunoreactivity was attributed to the beneficial effect of higher doses of SCFAs (30 mg/kg and 100 mg/kg) (Figure 5M,N for SP; Figure 5P,Q for SB), maintaining intestinal integrity following NTG-induced migraine. These results were confirmed by the ELISA kit (Figure 5S,T, respectively).

### 3.6. SCFA Administration Contributes to Decreased Neurotrophin Intestinal Immunoreactivity following NTG-Induced Migraine

**Figure 6:**
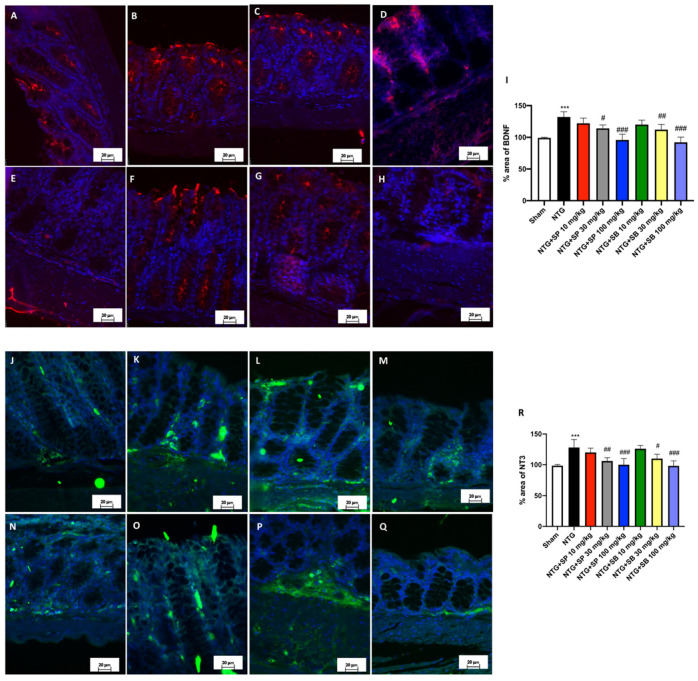
SCFA treatments decrease NT expression in the intestine following NTG injection. Positive NTs immunostaining is found in NTG-injected mice (**B**,**I**;**K**,**R**) compared to the sham animals (**A**,**I**;**J**,**R**). SCFAs of 30 mg/kg treatments (**D**,**G**,**M**,**P**), but most of all SCFAs of 100 mg/kg treatments (**E**,**N**,**H**,**Q**), reduce this positive staining. Mice treated with 10 mg/kg of SCFAs do not show any significant reduction in BDNF and NT expressions (**C**,**F**,**L**,**O**). Data are representative of at least three independent experiments; one-way ANOVA test. *** *p* < 0.001 vs. sham; **^#^** *p* < 0.05 vs. NTG; ^##^ *p* < 0.01 vs. NTG; ^###^ *p* < 0.001 vs. NTG. *N* = 10 mice/group for each technique.

Since NTs, known for their involvement in the regeneration and development of SNC, are overexpressed during a pathophysiological alteration in the gut, including Irritable Bowel Disease (IBS) and colitis [36], we investigated the Brain-Derived Nerve growth Factor (BDNF) and Neurotrophin-3 (NT-3) expressions in the intestine following NTG injection in mice. BDNF-like immunoreactivity was abundant in the mucosal epithelial cells of NTG-induced migraine mice compared to the sham group (Figure 6A,B, respectively). Quantification of the percentage area revealed that the expression of BDNF in the intestine was significantly attenuated by higher doses of SCFAs (both 30 mg/kg and 100 mg/kg) (Figure 6D,E for SP; Figure 6G,H for SB). However, a low dose of SFCAs did not demonstrate an important difference (Figure 6C,F for SP and SB, respectively). With further analysis of NTG-induced migraine mice on NT-3 immunoreactivity, no significant difference was found between NTG-injected mice and mice treated with 10 mg/kg of SCFAs (Figure 6L,O for SP and SB, respectively). NT-3 intestinal immunoreactivity was restored approximately to the basal levels by higher doses of SCFAs (30 mg/kg and 100 mg/kg) (Figure 6M,N for SP; Figure 6P,Q for SB). Tissue evaluation for neurotrophins in the intestinal tissue denoted that an axis between CNS-inflammatory-activated response following NTG-induced migraine and the intestinal functionality exists and could be simultaneously targeted by SCFAs.

### 3.7. Neuronal Nitric Oxide Production Is Downregulated following SCFA Administration in NTG-Injected Mice

**Figure 7:**
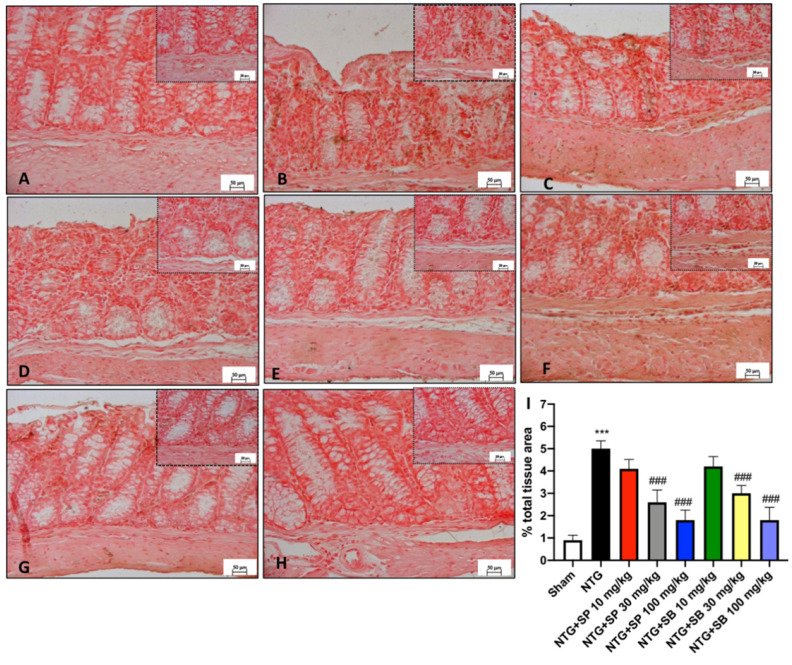
Effect of SCFA administration on nNOS expression in the intestine of NTG-injected mice. A marked positive staining of nNOS is detected in NTG mice (**B**,**I**) compared to the sham group (**A**,**I**). nNOS expression is significantly reduced in SCFA-treated animals at the two highest doses (**D**,**E**,**G**,**H**,**I**). Treatment with SCFAs of 10 mg/kg do not show any considerable reduction of nNOS expression (**C**,**F**,**I**). Data are representative of at least three independent experiments; one-way ANOVA test. *** *p* < 0.001 vs. sham; ^###^ *p* < 0.001 vs. NTG. *N* = 10 mice/group for each technique.

Nitric oxide (NO) release in response to nerve stimulation has been highlighted as an important player in different physiopathological conditions, including those of the mesenteric plexus [37]. Thus, to explore the production of NO and the maintenance of the enteric neurons’ health in mouse intestinal tissue, neuronal Nitric Oxide (nNOS) immunostaining was performed. In control mice, IHC revealed a basal positivity of the intestinal cells for nNOS (Figure 7A,I) compared to a significant increase in NTG-induced mice (Figure 7B,I), whereas the intestinal tissue sections from NTG mice treated with 10 mg/kg of SCFAs showed comparable expressions of nNOS to NTG-injected mice (Figure 7C,F,I). However, nNOS immunopositivity was found to decrease in both SP and SB at the higher doses of 30 mg/kg and 100 mg/kg (Figure 7D,E,G–I), helping to attenuate NO synthesis and release through the intestinal tissue layers following uncontrolled release due to activation of the neuroinflammatory cascade.

### 3.8. SCFA Treatments Modulate Proinflammatory Mediators following NTG-Induced Migraine

**Figure 8:**
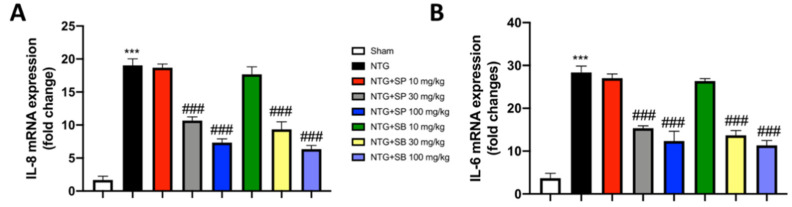
SCFA treatments decrease interleukin mRNA expression. NTG-injected mice show a significant increase in Il-6 and IL-8 mRNA expression. SCFAs of 30 mg/kg and 100 mg/kg decrease both interleukins expression following NTG administration ((**A**,**B**), respectively). Data are representative of at least three independent experiments; one-way ANOVA test. *** *p* < 0.001 vs. sham; ^###^ *p* < 0.001 vs. NTG. *N* = 10 mice/group for each technique.

Considerable clinical evidence [38,39] suggests that IL-6 and IL-8 are mainly involved in pain and in mediating neuroinflammation associated with migraine headaches. Therefore, we estimated the levels of both interleukins by RT-qPCR. A significant increase in both IL-6 and IL-8 mRNA expression levels was observed in NTG-injected mice compared to sham animals. Treatments with SCFAs at the two highest doses importantly reduced the mRNA expression for both cytokines, while SCFAs of 10 mg/kg did not show significant effects (Figure 8A,B).

## 4. Discussion

The overarching hypothesis for migraine pathophysiology describes it as a disorder of the pain-modulating system, caused by disruptions of the normal neural networks across the CNS and afferent neurons from these to peripheral system networks, including the enteric system [40]. There are many drugs used to treat migraine attacks including NSAIDs, which inhibit Prostaglandins (PGE) production, and triptans, stimulating the serotonin receptor 5-HT, principally used for the treatment of severe migraine attacks or those that do not respond to NSAIDs [41]. Both are considered as the first-line choice for episodic headaches, but additional treatments for migraine-caused symptoms are still needed. Here, a model of migraine induced by NTG injection was used to exhibit that SCFAs could alleviate short-term activation of the inflammatory cascade spreading in the brain. Current research has demonstrated that the examination of NTG-induced hyperalgesia and photophobia in vivo consists of substantial alterations in mice behaviors due to an important activation of the TGVS, reflecting an allodynic response activation to a painful stimulus [30]. At 4 h, the evident deficiencies observed in mice as their time in the dark chamber increased were importantly changed under the SCFA treatments. Moreover, the behavioral parameters of pain showed that the induced and raised hyperalgesia in NTG-induced migraine mice were modulated by the SP and SB treatments, showing that SCFAs could regulate pain and modulate the sensitization of a large number of nociceptive nerve fibers that originate in the trigeminal ganglion area. Previously, it was reported that nociceptive neurons depart from the SpV area, from which most of the headache attack symptoms originate [30]. The most remarkable finding of the study was that a single oral administration of both SP and SB prior to NTG injection was enough to markedly restore SpV neuronal degeneration, as well as the tissue architecture. Since vasodilatation and mast cell degranulation are considered important events during migraine [42], here, we confirmed the anti-inflammatory properties of SCFAs blocking the neuroinflammatory process of the CNS, similar to current treatments such as sumatriptan, through the decrease of pro-inflammatory mediator levels such as COX2 and iNOS.

Given that neuroinflammation and the gut functionality correlation are well identified as the gut–brain axis, here, we suggest that a potential and novel relationship between the activated inflammatory response of the CNS and the gut environment exists even during migraine onset and that SCFAs could counteract the correlated dysfunctionalities of the Gastrointestinal (GI) tract such as diarrhea, constipation, and gastroesophageal reflux (GERD) [9]. Our results clearly showed that intestinal integrity of mice markedly declined following NTG injection. In particular, we assessed that NTG exposure, while reproducing migraine pathology in mice, provokes intestinal mucosa collapse and disturbs gut equilibrium, confirming the possibility of a causal relation between neuroinflammation and changes in the gut environment [43].

Moreover, the altered intestinal tissue structure is highly correlated with the spreading of several markers of inflammation across the intestine layers [44]. In fact, the release of soluble pro-inflammatory factors in the intestine including TNF and IL-1β suggests small afferent fibers’ activation through the ENS [45]. In fact, the ENS is considered independent and in contrast with the Peripheral Nervous System (PNS), due to its ability to self-modulate a vast number of neurons including enteric astrocytes and the network of ganglia laid out along the gut. Therefore, the determination of the molecular mechanisms that regulate the responsiveness of the enteric system to the nerve growth factors is chief to understand their alterations within the ENS, providing a valid linkage between the gut environment and neuroinflammatory diseases, including migraine [46]. This possibility is also likely and supported by our findings because nerve process proliferation seems to increase Neurotrophins’ (NTs) release in intestinal epithelial cells [47]. Thus, considering that the physiological changes highlighted as a deregulated release of neurotransmitters in the ENS and their defective binding to the receptors are evident following migraine, which might be considered as crosstalk between the CNS and mucosal innervation, we demonstrated that the BDNF and NT-3 expression was significantly decreased in SCFA-treated mice, suggesting that SP and SB could accelerate mucosal recovery following intestinal compromise due to the inflammatory process activation in the brain.

Another approach to validate gut–brain axis involvement in migraine is through the investigation of the production of Nitric Oxide (NO), which is directly released in the brain following the excitation and activation of trigeminal neurons [48]. Moreover, it is also possible that an indirect mechanism of NO release could affect the enteric system when it crosses the brain blood barrier and comes to the intestine [49]. Here, we give the reasonable fact that NO synthesis increased following NTG-induced migraine, in its neuronal form, nNOS, which might be released under CNS control and spread along the peripheral nervous system including the ENS, while the SCFAs’ effect decreased NO synthesis and release through the intestinal tissue layers, lacking the activation of the neuroinflammatory cascade.

Furthermore, clinical studies elucidated the role of IL-6 and IL-8, which appeared significantly more expressed in patients with migraine than in healthy subjects [38,39].

Therefore, considering their involvement in pain induction and in the inflammatory mechanisms underlying migraine attacks, their management could represent an important objective in the migraine therapeutic approach.

The results obtained from the present study showed that the administrations of SCFAs strongly decreased the expression of both interleukins, thus suggesting once again the excellent ability of SCFAs to counteract the inflammatory state induced by migraine.

## 5. Conclusions

In conclusion, it is fair to say that SCFAs, injected orally, markedly act as modulators of the inflammation in the brain that occurs in migraine pathology, as well as strong modulators of the activation of the peripheral nervous fibers of the enteric system, reducing intestinal alteration.

## Data Availability

All of the results were generated and included in this study.
